# Retraction: AnnexinA5 might suppress the phenotype of human gastric cancer cells via ERK pathway

**DOI:** 10.3389/fonc.2023.1294881

**Published:** 2023-09-28

**Authors:** Frontiers Editorial Office

The journal retracts the May 12, 2021, article cited above.

Following publication, concerns were raised regarding the integrity of the images in the published figures. The authors failed to provide a satisfactory explanation during the investigation, which was conducted in accordance with Frontiers’ policies. Given the concerns about the validity of the data, the editors no longer have confidence in the findings presented in the article.

This retraction was approved by the Chief Editors of Frontiers in Oncology and the Chief Executive Editor of Frontiers.

The authors agreed with this retraction.

